# Theoretical Modelling, Experimental Testing and Simulation Analysis of Thermal Properties for Green Building-Insulation Materials

**DOI:** 10.3390/polym17030340

**Published:** 2025-01-26

**Authors:** Figen Balo

**Affiliations:** Department of METE, Engineering Faculty, Firat University, 23119 Elazig, Turkey; figenbalo@gmail.com

**Keywords:** epoxidized sesame oil, green building, renewable materials, ANSYS software, energy efficiency

## Abstract

In this study, 45 alternative green materials for building walls were experimentally produced, utilizing renewable (epoxidized sesame oil), natural (clay), and waste (Seyitömer fly ash) resources. These materials were evaluated based on key technical properties such as mass, tensile-compressive strength, and thermal conductivity, all of which are essential for construction and insulation applications. Subsequently, theoretical modeling was conducted for the material coded SE45, which demonstrated the lowest thermal conductivity. Through mathematical calculations, the theoretical thermal conductivity value was determined with a deviation of +5.88%. Furthermore, 48 alternative scenarios were designed for three different building envelope types (internally insulated, externally insulated, and sandwich), using commonly used building insulation materials alongside the sesame oil-based green material with the lowest thermal conductivity (SE45). Energy performance evaluations were conducted by analyzing temperature distributions along the walls of all designed scenarios using ANSYS simulations under the climatic conditions of Ankara, Turkey.

## 1. Introduction

In recent years, the construction and building industries have witnessed a growing shift toward sustainable materials to address the increasing global focus on energy efficiency, environmental protection, and resource conservation. The use of green materials, such as renewable and biodegradable resources, plays a crucial role in reducing the carbon footprint of buildings, enhancing their energy efficiency, and contributing to a healthier living environment. Incorporating these sustainable materials into building designs has become essential to meeting the demands of modern eco-friendly construction practices. The ability to modify plant oils chemically, such as through the process of epoxidation, opens new pathways for utilizing these materials in sustainable applications, including the development of green building-insulation materials, which are essential for modern eco-friendly construction practices.

Due to the anticipated decrease in oil reserves and the rise in ecological concerns, the use of sustainable raw materials in the polymer sector has been actively advancing [1]. Among these, plant oils stand out as one of the most accessible, cost-effective, and promising types of sustainable resources. Their lower toxicity and biodegradability further enhance their appeal as raw material for the polymer industry [2,3]. Plant oils primarily consist of fatty acids and triacylglycerols (glycerol tri-esters or triglycerides). The presence of double bonds in the hydrocarbon chains of the esters in plant oils enables chemical modifications, thereby broadening their range of applications [4,5]. The free fatty acid content is an important parameter in characterizing plant oils. More than a thousand types of fatty acids have been defined, but only approximately 20 are present in significant amounts in plant oils. Fatty acids make up 94–96% of the total mass of triglyceride molecules. Their carbon chain length varies between 22 and 14 C-atoms (with C18 and C16 being the most common), and they can have three to zero double bonds per carbon-carbon bond [6]. Table 1 lists the fatty acids with the highest ester content in plant-sourced oils [7].

The physicochemical features of vegetable oils are largely determined through their composition, including the fatty acid distribution, the position of double bonds, and the number of double bonds in the aliphatic chains, which define their suitability as sustainable raw materials for producing various substances through chemical reactions [8,9]. The range of applications for plant oils is significantly expanded through chemical modification, particularly via the introduction of double bonds in their structure. Vegetable oils are primarily used in the production of paints, coatings, agrochemicals, lubricants, biofuels, plasticizers, pharmaceuticals, and building materials, among other applications [10,11,12].

Epoxidized vegetable oils serve as versatile intermediates in a wide range of applications, including stabilizing compounds, polymer plasticizers, plastic additives, lubricant components, and polyols for urethane foam production [13,14,15], as well as in products such as medications, cosmetics, adhesives, elastomers, composite materials, surface coatings, and plasticizers [16,17]. Due to their numerous advantages, epoxidized vegetable oils, such as epoxidized camelina oil, have been employed as wood coatings, protecting materials from photodegradation and ultraviolet irradiation [18,19]. Additionally, epoxidized vegetable oils have been used as metal coating materials. For example, epoxidized soybean oil has been shown to reduce corrosion on steel [20], and it has been applied to starch films to enhance permeability and moisture resistance [21].

Epoxidized vegetable oils possess favorable chemical properties that make them highly suitable for polymer synthesis. The polymerization process involves the ring-opening of the epoxidized oil molecules [22]. Polyester polyols have been reported to be produced through the ring-opening of epoxidized oils such as soybean oil [23], palm oil [24], and broccoli seed oil [25]. Linseed and soybean oils have been observed to yield polyurethane foams with enhanced properties [26].

Due to their excellent thermal stability and low toxicity, epoxidized vegetable oils are also widely used in three-dimensional (3D) printing stereolithography. Epoxidized oils derived from sunflower, corn, soybean, tung, and linseed have demonstrated significant reactivity in 3D printing applications [27]. Hybrid resins of epoxidized plant oils, such as urethane-epoxidized soybean oil combined with acrylate, have exhibited improved mechanical strength, high detail resolution in prototypes, and superior surface finishes [23].

Furthermore, epoxidized vegetable oils have been shown to be valuable for four-dimensional (4D) printing. Applications of 4D printing span various fields, including optics, electronics, and medicine [28]. Sustainable materials, such as soybean oil epoxidized acrylate, have been documented for use in printing technologies [22].

Balo et al. developed materials with diverse technical properties suitable for construction and insulation applications using seven different oils. These materials were created through specific thermal processes, incorporating organic or waste materials such as clay, fly ash, perlite, and natural fibers [29,30,31,32,33,34,35]. The research on composite materials sourced from plant oils are given in Table 2.

In this study, green building insulation materials were produced using epoxidized sesame oil, fly ash, and clay. The performance of these materials, particularly in terms of insulation, was experimentally tested by evaluating key properties such as mass, compressive strength, tensile strength, and thermal conductivity coefficient. Subsequently, a mathematical model was developed to represent the experimentally determined minimum value of the thermal conductivity coefficient. The deviation between the theoretical predictions and the experimental results was calculated and analyzed. Finally, the thermal distribution characteristics of the material with the lowest thermal conductivity coefficient (SE45) were examined when applied to the walls of a sample building located in Ankara, Turkey, using ANSYS 2022 R2 software. For comparative analysis, the SE45-coded material was assessed alongside commonly used building and insulation materials under various environmental conditions using ANSYS simulations.

## 2. Experimental Study on the Manufacture of Materials Using Epoxidized Sesame Oil

### 2.1. Materials Used in the Experiments

One of the locally produced vegetable oils is sesame oil, which contains unsaturated bonds and is similar in composition to sunflower and olive oils. Sesame oil, in its unprocessed form, is a cheap oil that is widely produced in the markets. As a cooking oil suitable for food consumption, sesame oil is commonly used in small amounts in halva and other confections. Due to the large quantities produced, there have been attempts to transesterify sesame oil into biodiesel fuel [48]. Due to its high yield and competitive pricing compared to other oils, sesame oil is a popular choice for transesterification into biodiesel fuel [49,50]. Roasted sesame oil is particularly rich in tocopherols, sterols, and various unsaponifiables, including phenolic compounds and terpene alcohols. This has led to its use in creams and ointments, as well as as a natural antioxidant for bio-based diesel fuel [51,52]. After saponification, the unsaponifiable substance can be removed at room temperature. Therefore, the chemical composition of sesame oil makes it suitable for various applications. By utilizing the polymer peroxide in ethylene glycol polymerization to obtain sesame oil-based polyols, Gryglewicz first suggested that biopolyesters have the advantage of mimicking numerous features of the extracellular matrix and can be polymerized through peroxide interactions [53]. Sesame oil’s polyols and polyesters can serve as substitutes for synthetic materials [54,55,56]. Although there are many studies on sesame oil, there is no study in the literature on the production of materials for construction industry applications with epoxidized sesame oil. For this reason, this study, which integrates experimental research, mathematical modeling, and software-assisted analysis of building insulation materials designed by epoxidized sesame oil, represent novel research compared to all previously published studies. The fatty acid composition of selected vegetable oils, including sesame oil, are given in Table 3 [57].

Technical properties of sesame oil are summarized in Table 4 [58,59].

The disadvantages and advantages of various epoxidation strategies are presented in Table 5 [7].

Fly ash, a non-hazardous byproduct, is generated during the combustion of pulverized coal in coal-fired power plants for energy production. On its own, fly ash has limited advantages and is often collected and disposed of in landfills or stored at coal-fired power plants. However, fly ash has significant utility in the commercial and industrial sectors, primarily for enhancing the workability and durability of concrete mixes. Incorporating fly ash into concrete can mitigate its environmental risks and reduce its potential harm to humans, animals, and ecosystems. Fly ash is classified into two main categories: Class F and Class C. Class F fly ash, characterized by particulates coated with molten glass, enhances resistance to alkali-aggregate reactions and sulfate attacks while reducing the risk of concrete expansion. On the other hand, Class C fly ash, with its higher calcium oxide content, is more effective in strengthening structural concrete. In addition to its use in concrete, fly ash is employed as a filler in adhesives, paints, and metal and plastic composites. It is also widely used in road construction as structural fill and serves as a raw material for manufacturing plaster, Portland cement, ceramic tiles, bricks, ready-mix concrete, and other construction materials. Fly ash can be incorporated into products such as concrete bricks, grout fill, concrete pipes, wallboard, and hot-mix asphalt, further demonstrating its versatility in building applications [60].

Clay is an exceptionally versatile material with applications across various industries. In the construction sector, it is widely used for the production of lightweight blocks, concrete, precast components, and as structural backfill against foundations [61]. Moreover, clay has demonstrated significant potential in eliminating a range of pollutants commonly found in agricultural wastewater, including chemical O_2_ demand, total nitrogen, polyphenols, suspended solids, pesticides, and pharmaceuticals [62]. The properties of clay may vary depending on its producer or intended application. The characteristics of the fly ash and clay used in this study are presented in Table 6, while the properties of epoxidized sesame oil are detailed in Table 7. The technical specifications of the epoxidized plant oils were obtained from informational catalogs provided by their respective manufacturers.

### 2.2. Experimental Study

#### 2.2.1. The Prepare of 45 Different Samples

In this study, epoxidized sesame oil, fly ash, and clay were utilized to produce materials designed to provide insulation properties in buildings. The weight ratios of the materials used were determined based on preliminary studies. Fly ash and clay were incorporated at weight ratios of 30%, 40%, 50%, 60%, and 70%. Epoxidized sesame oil was used as a binder, mixed with fly ash and clay at weight ratios of 30%, 35%, and 40%. Samples were prepared by pouring the mixture into molds in the form of 100 mm × 100 mm × 100 mm cubes and 150 mm × 60 mm × 20 mm rectangular prisms. The samples underwent a pre-drying process at 95 °C, as determined from preliminary trials. Following this, they were placed in an oven at 3 different temperatures (185 °C, 165 °C, and 145 °C) for specific time intervals, allowing them to be prepared for experiments. Burning occurred in the samples at temperatures above 185 °C.

Compressive strength tests were conducted using cube samples, while thermal conductivity coefficients and mass values were determined using rectangular prism-shaped specimens. The sample codes, which correspond to the material proportions and applied temperatures of the 45 different samples prepared for the experiments, are displayed in Table 8. It has simplified the sample codes in the table to show only the numbers, with each number prefixed by ‘SE’ for consistency.

The mass, tensile strength, thermal conductivity, and compressive strength values of the obtained samples were determined experimentally. The compliance of these values with TS (Turkish Standards) and ASTM (American Society for Testing and Materials) standards were investigated to assess the material’s usability.

#### 2.2.2. Experimental Test Methodology

The characteristic of a material that defines its working temperature range is referred to as thermal conductivity, making it an essential factor to consider when addressing steady-state heat transfer problems. The hot-wire method has recently emerged as the preferred technique for determining the thermal properties of materials. This approach is particularly advantageous because, unlike calorimetric methods, it allows for the measurement of thermal conductivity at ambient temperatures. The hot-wire method has been applied extensively to determine the thermal characteristics of polymers and is recognized as an accurate and efficient method for measuring the thermal conductivity of ceramic materials [63,64]. In this investigation, the thermal conductivity coefficient of the produced materials is measured using the Shotherm-QTM gauge (Showa Denko K.K., Tokyo, Japan), which operates in accordance with the hot-wire technique described in DIN 51046. The measuring intervals range from 10 to 0.02 W/mK, and its sensitivity is reported to be +5% ± 1 digit. In this approach, two samples are sandwiched between the Cr-Ni hot-wire and the NiCr/Ni thermal element, which contains solder in the center. One sample’s thermal conductivity coefficient is known, while the other requires further investigation. According to Equation (1) [65], the heat conductivity coefficient can be determined as:

(1) $$k=K\frac{l^{2}ln(t_{2}-t_{1})}{V_{2}-V_{1}}-H$$

In this case, *H* and *K* are constants specific to the Shoterm-QTM apparatus, with values of 33·10^−3^ and 252·10^−4^, respectively. For each sample, measurements are taken three times at three distinct locations, and the mean of these nine thermal conductivity (*k*) values is used to determine the overall thermal conductivity coefficient.

The TS 699 standards [66] are applied for mass determination, oven-dried compressive strength testing, and surface abrasion loss (Bohme) testing of the samples. The equations provided in TS 699 are employed to evaluate these test results.

The compressive test is one of the most critical evaluations for determining the engineering quality of building materials. It measures the maximum load that a material can withstand under compression, allowing for quality control and performance comparison. For compressive strength testing, a Beskom brand test machine (Besmak Electronic, Ankara, Turkey) is used. This apparatus can handle pressures up to 200 tons per minute. The 10 cm × 10 cm × 10 cm cubic samples undergo compressive testing at a 5 mm/min crosshead speed. The results are automatically retrieved via a computer linked to the testing device.

The tensile strength of the samples is calculated using Equation (2) [67], where the compressive strength values, obtained at various temperatures, are used to compute the tensile strength.

(2) $$F_{\mathrm{Pulling}}=0.35\sqrt{F_{compressive}}$$

## 3. Results and Discussion

### 3.1. Test Results of Samples Obtained with Experimental Study

**Figure 1:**
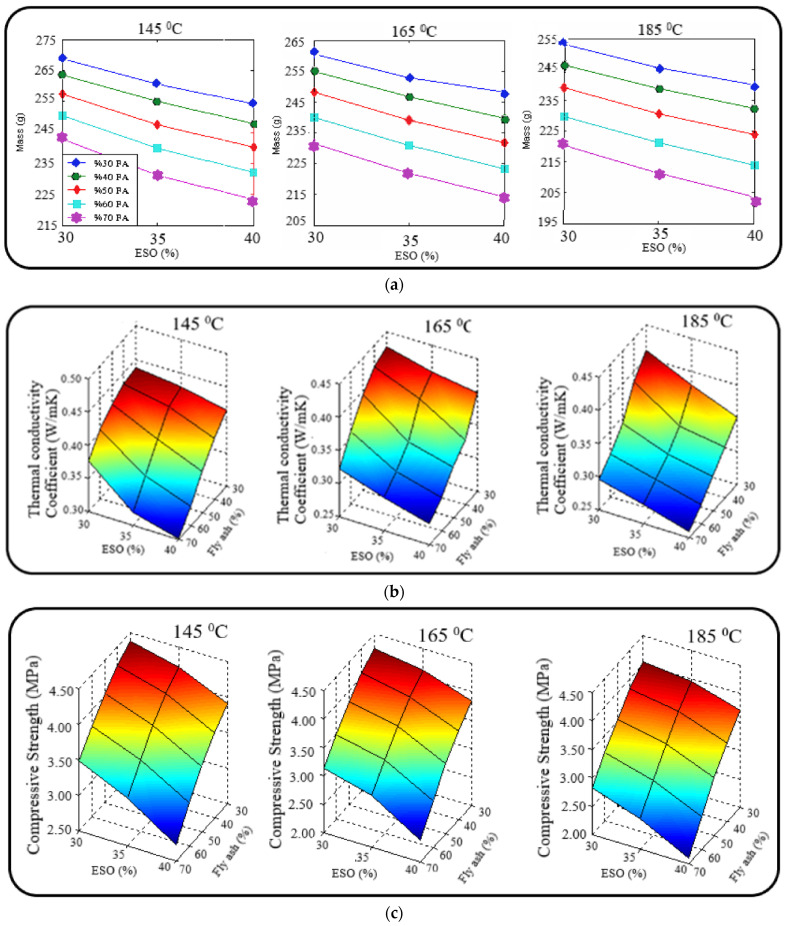

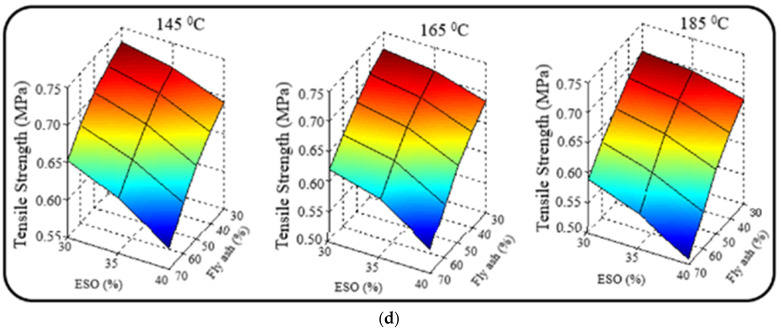
The experimental test results of produced samples with epoxidized sesame oil, clay, and fly ash (**a**) Mass (**b**) Thermal conductivity coefficient (**c**) Compressive strength (**d**) Tensile strength.

Mass is one of the key characteristics that influences many physical properties of lightweight materials. The change in mass values of specimens produced from ESO, fly ash, and clay at process temperatures of 145 °C, 165 °C, and 185 °C is illustrated in Figure 1a. When samples processed in the kiln, with an increasing amount of ESO in their structure, were examined at constant volume, a significant decrease in mass values was observed. At a process temperature of 185 °C, the mass values for samples containing 70% fly ash and 30% clay, with 30%, 35%, and 40% ESO, were 215.46 g, 206.10 g, and 198.72 g, respectively. This phenomenon can be attributed to the porosity that develops in the samples with an increasing amount of ESO in their structure, which was also evident when analyzing the heat transfer coefficients. When the process temperature was 165 °C, the mass values of the samples increased by 4.49%, 7.01%, and 10.6%, whereas at 145 °C, the increases were 6.30%, 10.27%, and 17.18%, respectively. The more significant decreases in mass values at higher temperatures suggest that elevated temperatures contribute to the formation of porous structures. Porous materials offer several advantages, including low thermal conductivity, reduced mass, and enhanced insulation capability. The minimum mass value of 198.61 g was obtained with sample SE45, made from 70% fly ash, 30% clay, and 40% ESO at a process temperature of 185 °C, while the maximum mass value of 262.39 g was observed with sample SE1, made from 30% fly ash, 70% clay, and 35% ESO at a process temperature of 145 °C. It was observed that the mass values of the produced materials increased as the quantity of fly ash decreased in the samples. When the quantity of fly ash in the SE1-coded sample was increased by 40%, 50%, 60%, or 70%, the mass values decreased by 0.32%, 0.94%, 2.03%, and 3.73%, respectively. It can be concluded that increasing the amount of fly ash in the structure leads to lower mass values, making the structure more porous. This is because the porous and amorphous structure of fly ash is more easily transferred into the sample matrix as the amount of fly ash increases.

The change in the coefficient of thermal conductivity values of the produced materials treated at 145 °C, 165 °C, and 185 °C, according to the varying amounts of fly ash, clay, and ESO in their structure, is shown in Figure 1b. It was observed that the coefficient of thermal conductivity values decreased as the amount of ESO in the sample structure increased. The minimum coefficient of thermal conductivity values was found at the highest ESO content (0.256 W/mK with 40% ESO), while the highest values were recorded at the lowest ESO content (30%). At the treatment temperature of 145 °C, the thermal conductivity coefficient values of the samples prepared with 30%, 35%, and 40% ESO at a 30% fly ash/70% clay ratio show a decrease of 6.33%, 10.39%, and 17.35%, compared to the materials produced at 165 °C and 185 °C with similar material ratios. It was observed that the coefficient of thermal conductivity values of the samples decreased as the process temperature increased. The highest coefficient of thermal conductivity was found in samples with 30% fly ash content, while the lowest value was recorded at 70% fly ash content. The minimum coefficient of thermal conductivity (0.256 W/mK) was achieved with sample SE45, treated at 185 °C with a material composition of 70% fly ash, 30% clay, and 40% ESO. When the amount of fly ash in sample SE45 was reduced by 60%, 50%, 40%, or 30%, the thermal conductivity coefficient values increased by 32.92%, 44.96%, 51.42%, and 54.65%, respectively. This trend can be attributed to the porosity effect present in the natural structure of fly ash, which becomes more pronounced as its content in the sample increases. Fly ash is a partially amorphous and porous material with low density. Previous studies have demonstrated that low thermal conductivity values can be achieved with materials exhibiting an amorphous and porous structure [68]. Therefore, it is an expected result that the coefficient of thermal conductivity for the fly ash, clay, and ESO samples decreases with increasing fly ash content. Thermal insulation materials are characterized by a coefficient of thermal conductivity of less than 0.065 W/mK, as specified by TS 825 and EN Standards [69]. The coefficient of thermal conductivity of the SE45-coded material, with the lowest thermal conductivity value obtained through experimental research, was found to be 0.256 W/mK. Therefore, this material can be considered a green building material with excellent insulating properties.

The change in compressive strength values of specimens treated at 145 °C, 165 °C, and 185 °C, depending on the amount of ESO, fly ash, and clay in the specimens, is displayed in Figure 1c. The compressive strength values of the ESO, fly ash, and clay specimens vary between 12.69 MPa and 8.63 MPa. The maximum compressive strength was achieved from the specimen coded SE1, made from 30% fly ash, 70% clay, and 30% ESO, treated at 145 °C. The compressive strength value was recorded as 12.60 MPa, which increased slightly to 12.52 MPa when the ESO ratio was adjusted to 35% and 45%, respectively. It was found that as the fly ash content in the structure increased, meaning as the quantity of clay decreased, the compressive strength values of the specimens decreased. When the clay content in the specimen coded SE1 was reduced by 60%, 50%, 40%, or 30%, the compressive strength values decreased by 0.45%, 1.30%, 2.79%, and 5.49%, respectively. This indicates that increasing the amount of fly ash in the material leads to a greater porosity structure, which, in turn, reduces the compressive strength of the sample. On the other hand, the presence of clay enhances the structural integrity, positively impacting the bonding of particles. When the compressive strength values were compared proportionally according to the process temperatures at which the samples were produced, it was observed that the highest compressive strength values were achieved at 145 °C, while the lowest compressive strength values were recorded at 185 °C.

The tensile strength values, calculated according to Equation (1), are presented in Figure 1d, demonstrating the relationship between tensile strength and compressive strength in materials produced from fly ash, clay, and ESO at process temperatures of 185 °C, 165 °C, and 145 °C. The SE1-coded sample, composed of 30% fly ash, 70% clay, and 30% ESO, exhibited the highest tensile strength of 1.240 MPa at 145 °C. In contrast, the SE45-coded sample, made from 70% fly ash, 30% clay, and 40% ESO, showed the lowest tensile strength of 1.028 MPa at 185 °C.

All experimental results indicate the development of porous structures and the formation of heat shields. As the amount of ESO in the sample structure increases, significant porosity is created, leading to a decrease in mass and thermal conductivity. These porous structures not only reduce mass but also enhance insulation properties, making the material ideal for thermal barrier applications. Furthermore, as the process temperature increases, these porous formations become more pronounced, contributing to better heat resistance. The samples produced with varying fly ash, clay, and ESO ratios show varying compressive and tensile strengths, influenced by the level of porosity. The presence of clay in the structure strengthens the bonding between particles, while the amorphous and porous nature of fly ash reduces compressive strength but improves thermal performance. Overall, these results demonstrate the potential of these materials to serve as effective thermal shields, combining low thermal conductivity with sufficient structural integrity.

### 3.2. Theoretical Calculation of Thermal Conductivity Coefficient in Porous Materials

Porous materials consist of particles and pores that come in various forms and sizes. Their properties are highly dependent on the characteristics and volumes of the materials that make up their structure. Analyzing the fundamental thermodynamic and hydrodynamic functions of porous building materials are more complex than applying these functions to non-porous materials. However, by investigating the elementary cell structure of the material through some assumptions, a clearer understanding of the entire structure can be achieved. Heat transfer in porous solid materials occurs through conduction (thermal conductivity), convection (heat convection), and radiation. To develop a theoretical model for calculating the heat conductivity coefficient of porous solid materials, the following assumptions are made, taking into account these thermal transfer mechanisms:

- The pore diameters of the fly ash used in this study are smaller than 1 mm. Thus,

$$Ra=Gr\mathrm{Pr}<10^{-3}$$

Consequently, it is assumed that natural thermal conductivity will not occur.

- Based on the Stefan-Boltzmann law, the thermal conductivity through radiation is calculated using the following equation:

(3) $$Q_{lş}=\varepsilon \sigma \left(T_{1}^{4}-T_{2}^{4}\right)$$

Since the pores’ wall temperatures will be very close to each of the others $\left(T_{1}≅T_{2}\right)$, thermal conductivity through radiation can be neglected.

- Although pore shapes are in irregular and complex geometrical forms, they are close to spheres. In order to be able to take the cross-section constant in the thermal flow direction, the assumption is that the pore cross sections are square.
- There is an assumption that moisture does not exist within the material structure.

The mean effective thermal conductivity of a heterogeneous structure, made of different materials with varying thermal conductivities, is referred to as the effective thermal conductivity (kef). The effective heat conductivity coefficient of porous solid materials is a function of various factors, such as the thermal conductivity of the gas inside the pores.

$$k_{ef}=f(\phi ,k_{s},k_{g},\rho )$$

**Figure 2:**
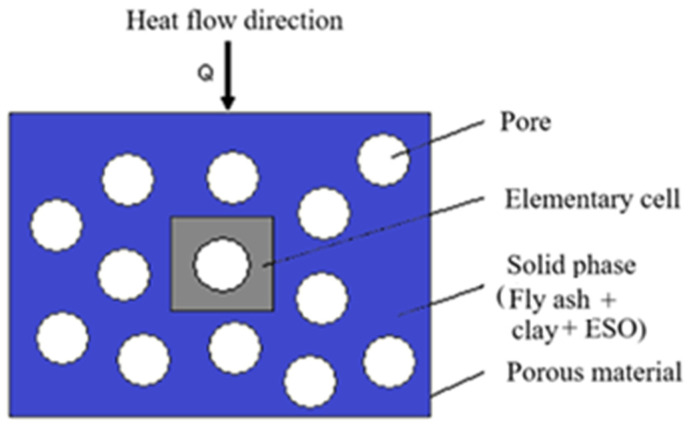
Physical model of the porous material.

In porous materials, the greater the mismatch between the pore shapes and known geometrical forms, the higher the observed porosity in the material. Pores are randomly distributed throughout the structure and predominantly take on a spherical form. Surrounding the pores is a solid matrix composed of clay, ash, and synthesized plant oil. A physical model representation is displayed in Figure 2. In this model, only one elementary cell is considered for these structures.

The thermal flow direction is oriented parallel to the vertical lines of the elementary cell. The one-dimensional heat conductivity expression, based on Fourier’s law of thermal conductivity, is:

(4) $$Q=-kA\left(dT/dx\right)$$

Taking the integral of this expression,

(5) $$Q=\frac{T_{sıc}-T_{soğ}}{\left(1/kA\right)L}$$

can be stated. (1/kA)L is the thermal resistance and represented by R. Thus, the expression below can be obtained:

$$Q\;R=T_{sıc}-T_{soğ}$$

Applying this equation to the elementary cell cross-section;

For cross-section number (1):

(6) $$T_{1}-T_{6}=Q_{1}{\;R}_{1K}=Q_{1}{\;R}_{1eş}$$

For cross-section number (2):

$$\begin{array}{cc} T_{1}-T_{2} & =Q_{2}\;R_{2K}\\ T_{2}-T_{5} & =Q_{2}\;R_{2K}\\ T_{5}-T_{6} & =Q_{2}\;R_{2K} \end{array}$$

Adding the expressions:

$$\begin{array}{l} T_{1}-T_{6}=Q_{2}(R_{2K}+R_{2U}+R_{2K})\\ T_{1}-T_{6}=Q_{2}(2R_{2K}+R_{2U}) \end{array}$$

(7) $$T_{1}-T_{6}=Q_{2}(2R_{eş})$$

For cross-section number (3):

$$T_{1}-T_{2}=Q_{3}R_{3K}\;T_{2}-T_{3}=Q_{3}R_{3U}\;T_{3}-T_{4}=Q_{3}R_{3G}\;T_{4}-T_{5}=Q_{3}R_{3U}\;T_{5}-T_{6}=Q_{3}R_{3K}$$

Adding the expressions:

$$\begin{array}{l} T_{1}-T_{6}=Q_{3}(R_{3K}+R_{3U}+R_{3G}+R_{3U}+R_{3K})\\ T_{1}-T_{6}=Q_{3}(2R_{3K}+2R_{3U}+R_{3G}) \end{array}$$

(8) $$T_{1}-T_{6}=Q_{3}R_{3eş}$$

**Figure 3:**
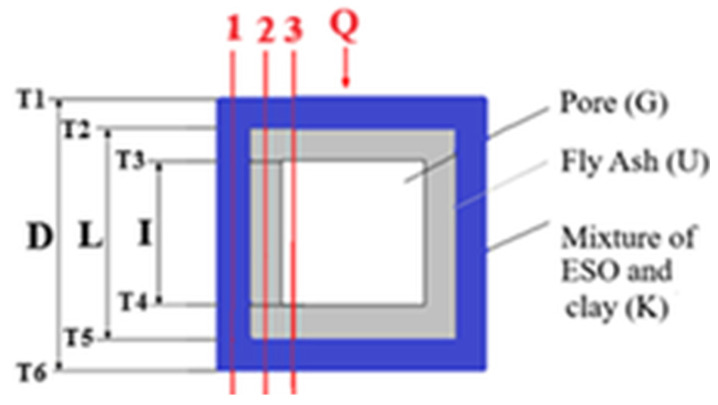
Cross-section of the elementary cell.

**Figure 4:**
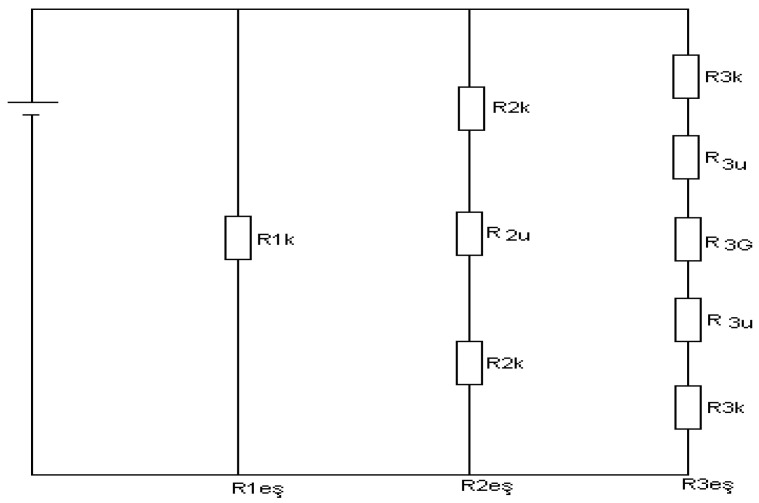
Electrical similarity circuit scheme of elementary cell components thermal resistances.

Equation (5) can be obtained. On the other hand, for the elementary cell as a whole,

(9) $$Q=k_{efs}A\frac{T_{1}-T_{6}}{D}=\frac{T_{1}-T_{6}}{D/k_{efs}A}=\frac{T_{1}-T_{6}}{R_{eş}}$$

can be written. Thermal amount going through the elementary cell provided in Figure 3 can be written as,

(10) $$Q=Q_{1}+Q_{2}+Q_{3}$$

because of its components being parallel to each other. Using Equations (3)–(6), and placing $Q_{1}$, $Q_{2}$, $Q_{3}$, and Q values in Equation (7)

(11) $$\frac{T_{1}-T_{6}}{R_{eş}}=\frac{T_{1}-T_{6}}{R_{1eş}}+\frac{T_{1}-T_{6}}{R_{2eş}}+\frac{T_{1}-T_{6}}{R_{3eş}}$$

can be obtained. Canceling out $\left(T_{1}-T_{6}\right)$ values on both sides of Equation (8), the following

(12) $$\frac{1}{R_{eş}}=\frac{1}{R_{1eş}}+\frac{1}{R_{2eş}}+\frac{1}{R_{3eş}}$$

expression can be written. This equation represents the expression for the parallel-connected resistances from the electrical analogy circuit depicted in Figure 4, corresponding to the elementary cell cross-section shown in Figure 3. The thermal resistances of the components within this elementary cell can be expressed as follows Table 9:

These resistances are defined as follows: the solid phase of the first component (clay + ESO), the solid phase of the second component (fly ash), and the resistance of the third component (pore). The equivalent resistances can be expressed as follows by summing the series-connected resistances of the electrical analogy circuit:

$$\begin{array}{l} R_{1eş}=R_{1K}\\ R_{2eş}=R_{2K}+R_{2U}\\ R_{3eş}=2R_{3K}+R_{3U}+R_{3G} \end{array}$$

Placing these equivalent resistances in Equation (9) and doing the necessary calculations, Equations (14) and (15) can be obtained.

(13) $$\frac{1}{R_{eş}}=\frac{1}{\frac{D}{k_{K}\left(D^{2}-L^{2}\right)}}+\frac{1}{\frac{\left(D-L\right)}{k_{K}\left(L^{2}-I^{2}\right)}+\frac{L}{k_{U}\left(L^{2}-I^{2}\right)}}+\frac{1}{\frac{\left(D-L\right)}{k_{K}I^{2}}+\frac{\left(L-I\right)}{k_{U}I^{2}}+\frac{I}{k_{G}I^{2}}}$$

(14) $$\frac{1}{R_{eş}}=\frac{k_{K}\left(D^{2}-L^{2}\right)}{D}+\frac{k_{K}k_{U}\left(L^{2}-I^{2}\right)}{k_{U}\left(D-L\right)+k_{K}L}+\frac{k_{K}k_{U}k_{G}I^{2}}{k_{U}k_{G}\left(D-L\right)+k_{K}k_{U}\left(L-I\right)+k_{K}k_{U}I}$$

Writing $R_{eş}$ expression in Equation (6) as in Equation (12),

(15) $$R_{eş}=\frac{D}{k_{efs}A}=\frac{1}{k_{efs}D^{2}}+\frac{1}{k_{efs}D}$$

Placing in (11) and doing the necessary calculations;

(16) $$k_{efs}D=\frac{1}{R_{eş}}=\frac{k_{K}\left(D^{2}-L^{2}\right)}{D}+\frac{k_{K}k_{U}\left(L^{2}-I^{2}\right)}{k_{U}\left(D-L\right)+k_{K}L}+\frac{k_{K}k_{U}k_{G}I^{2}}{k_{U}k_{G}\left(D-L\right)+k_{K}k_{G}\left(L-I\right)+k_{K}k_{U}I}$$

(17) $$k_{efs}=\frac{k_{K}\left(D^{2}-L^{2}\right)}{D^{2}}+\frac{k_{K}k_{U}\left(L^{2}-I^{2}\right)}{k_{U}D\left(D-L\right)+k_{K}DL}+\frac{k_{K}k_{U}k_{G}I^{2}}{k_{U}k_{G}D\left(D-L\right)+k_{K}k_{G}D\left(L-I\right)+k_{K}k_{U}DI}$$

(18) $$\begin{array}{ll} k_{efs}=k_{K}\left[1-{\left(L/D\right)}^{2}\right] & +\frac{k_{K}k_{U}\left(L^{2}-I^{2}\right)}{k_{U}D^{2}\left[1-\left(L/D\right)\right]+k_{K}D^{2}\left(L/D\right)}\\ & +\frac{k_{K}k_{U}k_{G}I^{2}}{k_{U}k_{G}D^{2}\left[1-\left(L/D\right)\right]+k_{K}k_{G}D^{2}\left[\left(L/I\right)/D\right]+k_{K}k_{U}D^{2}\left(I/D\right)} \end{array}$$

(19) $$\begin{array}{ll} k_{efs}=k_{K}\left[1-{\left(L/D\right)}^{2}\right] & +\frac{k_{K}k_{U}\left[{\left(L/D\right)}^{2}-{\left(I/D\right)}^{2}\right]}{k_{U}\left[1-\left(L/D\right)\right]+k_{K}\left(L/D\right)}\\ & +\frac{k_{K}k_{U}k_{G}{\left(I/D\right)}^{2}}{k_{U}k_{G}\left[1-\left(L/D\right)\right]+k_{K}k_{G}\left[\left(L/D\right)-(I/D)\right]+k_{K}k_{U}\left(L/D\right)} \end{array}$$

The above equations can be obtained.

$$L^{3}/D^{3}=Z\;(\mathrm{Mixture}\;\mathrm{ratio}=\mathrm{Volume}\;\mathrm{of}\;\mathrm{fly}\;\mathrm{ash}/\mathrm{Total})$$

$$I^{3}/D^{3}=\phi \;(\mathrm{Porosity}=\mathrm{Volume}\;\mathrm{of}\;\mathrm{pore}/\mathrm{Total}\;\mathrm{volume})$$

Placing the above values in Equation (16) and making the mathematical calculations, the expression resulting in the porous solid materials’ heat conductivity coefficient can be found as follows:

(20) $$\begin{array}{l} k_{efs}=k_{K}k_{U}k_{G}\left[\begin{array}{l} \frac{1}{k_{U}k_{G}}\left(1-Z^{2/3}\right)+\frac{\left(Z^{2/3}-\phi^{2/3}\right)}{k_{U}k_{G}\left(1-Z^{1/3}\right)+k_{K}k_{G}Z^{1/3}}\\ \;\;\;\;\;\;\;\;\;\;\;\;\;+\frac{\phi^{2/3}}{k_{U}k_{G}\left(1-Z^{1/3}\right)+k_{K}k_{G}\left(Z^{1/3}-\phi^{1/3}\right)+k_{K}k_{U}\phi^{1/3}} \end{array}\right] \end{array}$$

Equation (17) is formulated for porous solid materials. Thus, this equation cannot be used for the samples with mixed rates of ESO, clay, and fly ash that are not able to form a solid structure.

#### 3.2.1. Determining the Model Parameters with Experimental or Theoretical Methods

##### Porosity

Because they can resist the heat conductivity of the air inside the pore, porous materials can act as insulation. The coefficient of thermal conductivity of the material decreases in light of the increasing porosity rate. That is why the material porosity has an important effect on their heat conductivity.

The porous material’s porosity (ϕ) is the rate of its pore volume to the total volume, and it is a unit with no dimension.

ϕ=Volume of pores/Total volume

Showing the volume of pores ($V_{G}$) with the volume of the solid part $V_{K}$ and total volume V is:

(21) $$V_{G}+V_{K}=V$$

Dividing both sides of Equation (18) by the total volume, the following,

(22) $$\frac{V_{G}}{V}+\frac{V_{K}}{V}=1$$

equation can be obtained.

Leaving $V_{K}/V$ on the left-hand side and using porosity rate expression $V_{G}/V=\phi$ from the definition, the below,

(23) $$V_{K}/V=1-(V_{G}/V)=1-\phi$$

can be written. Sample mass,

$$\rho V=\rho_{FlyAsh}V_{FlyAsh}+\rho_{binder}V_{binder}$$

(24) $$\rho =(\rho_{FlyAsh}V_{FlyAsh}+\rho_{binder}V_{binder})/V$$

can be written as above.

V=Sample volume

$V_{K}=\mathrm{Volume}\;\mathrm{of}\;\mathrm{non-porous}\;\mathrm{sample}\;(\mathrm{volume}\;\mathrm{of}\;\mathrm{solid}\;\mathrm{part})$

Thus;

(25) $$V=V_{FlyAsh}+V_{binder}$$

(26) $$V_{FlyAsh}=V-V_{binder}$$

(27) $$V_{binder}=V_{K}-V_{FlyAsh/matrix}$$

writing Equation (27) into Equation (25), the following

(28) $$V_{FlyAsh}=V-(V_{K}-V_{FlyAsh/matrix})$$

equation can be obtained.

To determine the density of the porous sample, Equations (27) and (28) can be written within Equation (24) as follows,

$$\rho =\rho_{FlyAsh}(V-V_{K}+V_{FlyAsh/matrix})/V+\rho_{binder}(V_{K}-V_{FlyAsh/matrix})/V$$

doing the required calculations the subsequent

$$\rho =\rho_{FlyAsh}+(\rho_{bider}-\rho_{FlyAsh})\frac{V_{K}}{V}(1-\frac{V_{FlyAsh/matrix}}{V})$$

$Z=\frac{V_{FlyAsh/matrix}}{V_{K}}$ thus;

(29) $$\rho =\rho_{FlyAsh}+(\rho_{binder}-\rho_{FlyAsh})(1-\phi )(1-Z)$$

equation can be obtained.

Writing Equation (29) inside the $\phi =1-\frac{\rho }{\rho_{K}}$ equation, porosity value can be calculated as in the Equation (30) below:

$$\phi =\{\rho_{K}-[\rho_{FlyAsh}+(\rho_{binder}-\rho_{FlyAsh})(1-\phi )(1-Z)]\}/\rho_{K}$$

(30) $$\phi =\frac{\rho_{K}-\rho_{FlyAsh}-(\rho_{binder}-\rho_{FlyAsh})(1-Z)}{\rho_{K}-(\rho_{binder}-\rho_{FlyAsh})(1-Z)}$$

In this equation; $\rho_{binder}=\rho_{clay+ESO}$.

**Figure 5:**
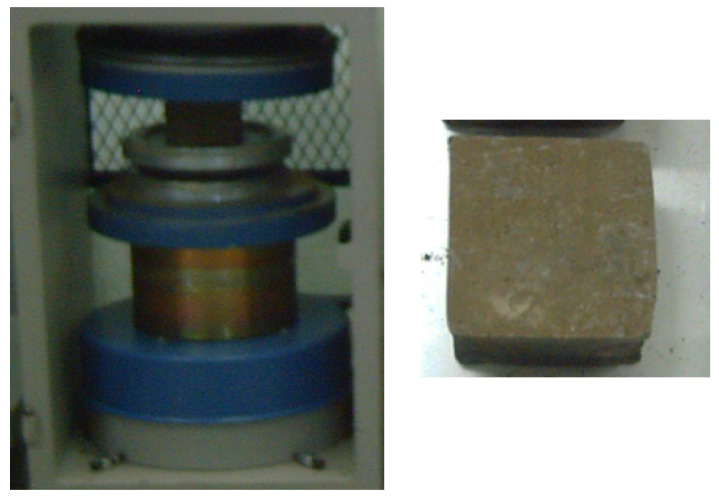
Porous structure observed in the samples after corrosion.

In this study, the sample (SE45) obtained from Seyitömer Thermal Power Plant, which contains 70% chimney bottom fly ash, 30% clay, and 40% epoxidized sesame oil, exhibit the lowest mass and the lowest thermal conductivity coefficient (indicating the highest porosity). Technical values of the fly ash and clay materials used in the experiments are displayed in Table 10 [70]. Porous structure observed in the samples after corrosion is given in Figure 5.

##### Volume and Weight Relationship in Ash-Binder Mixtures

Weight = density × volume × gravity acceleration.

To find out the weights of the ash and binder

$$\begin{array}{ll} WFlyAsh & =\rho_{FlyAsh}V_{FlyAsh}g\\ Wbinder & =\rho_{binder}V_{binder}g \end{array}$$

the above equations can be written. Dividing both sides of these equations,

(31) $$\frac{W_{FlyAsh}}{W_{binder}}=\frac{\rho_{FlyAsh}}{\rho_{binder}}\cdot \frac{V_{FlyAsh}}{V_{binder}}$$

the above expression can be obtained. Dividing the left-hand side of the equation by produced material weight (W) and dividing the right-hand side by the produced material volume (V) the below

$$\frac{W_{FlyAsh}}{W}=F=\mathrm{Ash}\;\mathrm{rate}\;\mathrm{of}\;\mathrm{weight}$$

$$\frac{W_{binder}}{W}=1-F=\mathrm{Ash}\;\mathrm{rate}\;\mathrm{of}\;\mathrm{binder}$$

$$\frac{V_{FlyAsh}}{V}=Z=\mathrm{Ash}\;\mathrm{rate}\;\mathrm{by}\;\mathrm{volume}$$

$$\frac{V_{binder}}{V}=1-Z=\mathrm{Ash}\;\mathrm{rate}\;\mathrm{by}\;\mathrm{binder}$$

transformations can be made. Placing these expressions within Equation (31) and leaving the Z value on the left-hand side the following

(32) $$Z=\frac{\rho_{\left(binder\right)}}{\rho_{\left(FlyAsh\right)}}=\frac{F/\left(1-F\right)}{1+\left(\rho_{\left(binder\right)}/\rho_{FlyAsh}\right)\left(F/\left(1-F\right)\right)}$$

equation can be obtained.

##### Thermal Conductivity Coefficients of Non-Porous Ash, Clay, and Binder

The density of the fly ash screened at 400 mesh (35.10–6 m) screens is measured as 2.10 g/cm^3^. This value is very close to the theoretically calculated density value of 2.142 g/cm^3^. Thus, it is classified as a non-porous ash. The thermal conductivity value of this fly ash is measured after mixing it with water, pouring it into a mold, compressing it under pressure, and drying it at 200C room temperature for 28 days. Similarly, the density of clay is measured to be 1.527 g/cm^3^ after screening it at a 400 mesh (35.10–6 m) screen. This value is also very close to the theoretically calculated density value of 1.177 g/cm^3^. Applying the same steps to the created sample with clay, the clay’s thermal conductivity is measured.

##### Thermal Conductivity Coefficients of the Gases Inside the Pore

Pores within the fly ash structure occur at 1200 °C oven temperature. It is observed that the rates of NO_2_ and CO_2_ gases are higher compared to those of other components. Thus, the gas inside the pores is assumed to be a mixture of NO_2_ and CO_2_ gases.

The heat conductivity coefficient of this gas mixture is computed as the mean coefficients of thermal conductivity of NO_2_ and CO_2_ gases [71,72].

k_G_ = (k_Co2_ + k_N2_)/2 = 0.0212 (W/mK) (33)

Thermal conductivity coefficients of the materials used in this study $k_{bağ}=k_{kil+EVO}$ using clay and epoxidized plant oils at determined rates and produced mixtures. These mixtures are poured into molds, and their density values are determined after going through the baking processes.

##### Quantitative Calculation of Coefficient of Thermal Conductivity of a Sample

Sample S27 is used for the quantitative solution. This sample is obtained through a mixture of Seyitömer chimney bottom fly ash, clay, and epoxidized soybean oil in a 1:1:1 ratio and baking first at 100 °C for 9 h, then at 185 °C for 8 h. The fly ash and binder ratios of the sample are as follows:

Weight percentages of the mixture: 33% fly ash/66% clay + ESO
Volume percentages of the mixture: 21.4% fly ash/78.6% clay + ESO

Using Equation (29), the volume percentage of the mixture is calculated to be:

$$Z=\frac{\rho_{binder}}{\rho_{\left(FlyAsh\right)}}\frac{F/\left(1-F\right)}{1+\left(\rho_{binder}/\rho_{\left(FlyAsh\right)}\right)\left(F/1-F\right)}$$

Using Equation (26), porosity value is calculated as follows:

$$\phi =\frac{\rho_{K}-\rho_{FlyAsh}+(\rho_{binder}-\rho_{FlyAsh})(1-Z)}{\rho_{K}-(\rho_{binder}-\rho_{FlyAsh})(1-Z)}$$

Placing Z and ϕ values calculated as above, values presented, along with the values provided below at the developed Equation (17), we obtain:

$$k_{G}=0.0194\;W/mK$$

$$\rho_{U}=2.10\;{g/cm}^{3}$$

$$\rho_{Umatrix}=1.04\;{g/cm}^{3}$$

$$k_{U}=2.142\;W/mK$$

$$\begin{array}{l} k_{efs}=k_{K}k_{U}k_{G}\left[\begin{array}{l} \frac{1}{k_{U}k_{G}}\left(1-Z^{2/3}\right)+\frac{\left(Z^{2/3}-\phi^{2/3}\right)}{k_{U}k_{G}\left(1-Z^{1/3}\right)+k_{K}k_{G}Z^{1/3}}\\ \;\;\;\;\;\;\;\;\;\;\;\;\;\;\;+\frac{\phi^{2/3}}{k_{U}k_{G}\left(1-Z^{1/3}\right)+k_{K}k_{G}\left(Z^{1/3}-\phi^{1/3}\right)+k_{K}k_{U}\phi^{1/3}} \end{array}\right] \end{array}$$

The experimental thermal conductivity coefficient of the material coded as SE45 is measured to be 0.256 W/mK. This value closely aligns with the heat conductivity coefficient calculated through the steps outlined in the theoretical model. The deviation between the experimentally obtained value and the value derived from mathematical calculations is found to be 5.88%. The k binder value used in the calculations represents the thermal conductivity coefficient measured after the sample, produced with a 40% clay-40% epoxidized sesame oil (ESO) mixture, undergoes all relevant thermal processing steps.

### 3.3. ANSYS Software Analysis

The identification of epoxidized sesame oil-based building insulation material as a study object, in alignment with green building principles, necessitates a thorough evaluation of the strategies to be employed.

**Figure 6:**
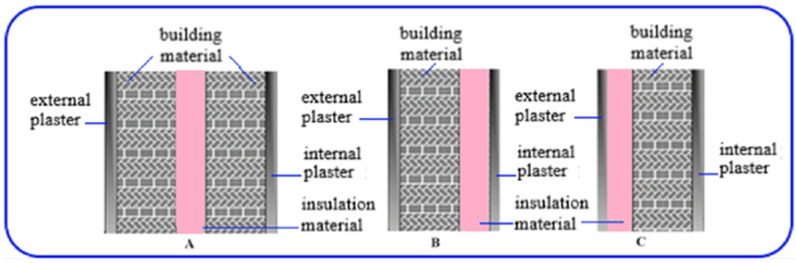
Wall structures evaluated with building exterior wall components. (**A**) Sandwich (**B**) Internally insulated (**C**) Externally insulated.

Another component of this study involved investigating the thermal distributions for a sample building wall in Ankara using ANSYS software. The thermophysical properties of four commonly used insulation materials—EPS, XPS, wool, and polyurethane—and four common building materials—aerated concrete, bims, brick, and SE4—were used as inputs in ANSYS. For comparison with conventional building materials, the material coded SE45, which exhibited the lowest thermal conductivity coefficient based on experimental tests using sesame oil, was selected. To provide a comprehensive comparison, 48 different alternative scenarios were created, encompassing three distinct wall structures: externally insulated, internally insulated, and sandwich walls. The evaluation of these wall structures with building exterior components is displayed in Figure 6. The design of alternative scenarios using the SE45-coded sample, alongside traditional building insulation materials, for the three different wall structures is provided in Table 11. The technical properties of the materials used in the ANSYS analysis are detailed in Table 12.

ANSYS analysis was conducted using various parameters, including the climate data for the city of Ankara, internal and external comfort design conditions, and the thermophysical properties of the materials. This analysis aimed to identify more energy-efficient designs by evaluating temperature distributions in building envelope designs in the Ankara province. The walls designed with the SE45-coded patterns were compared to alternative wall designs. The best and worst combinations for the three wall structures were determined. The comparative evaluations were illustrated through thermal distribution graphs generated using ANSYS analysis.

**Figure 7:**
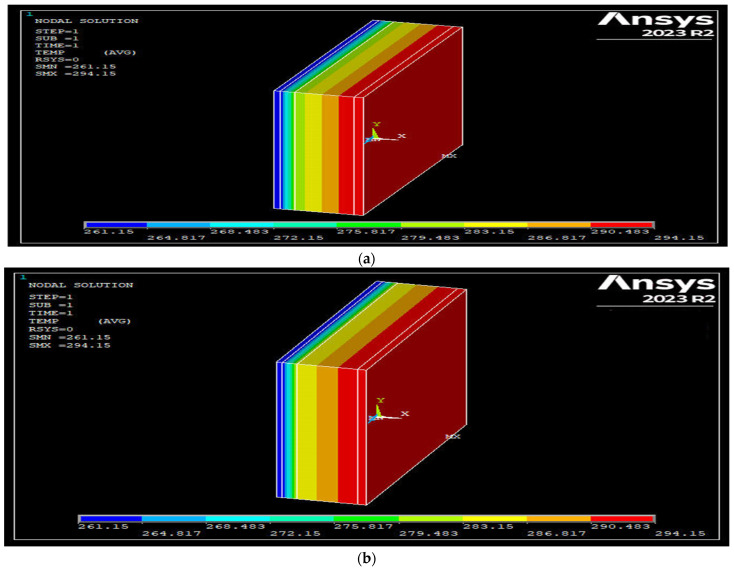
(**a**) Vectorial view of the best-performing building envelope among the alternative scenarios designed for Ankara province (Type 36). (**b**) Vectorial view of the best-performing building envelope obtained by ANSYS analysis using specimen SE45 (Type 48).

In the building envelope designed for the Ankara province, Type 36 emerged as the top-performing alternative among all wall types. This alternative was designed using aerated concrete as the construction material and polyurethane as the insulation material in an externally insulated wall structure. When the SE45-coded specimen was used as the construction material in the wall components, the alternative scenario with the best performance was designated as Type 48. Figure 7 presents the vectorial views of the wall temperature distributions obtained through ANSYS analysis for Type 36 and Type 48.

**Figure 8:**
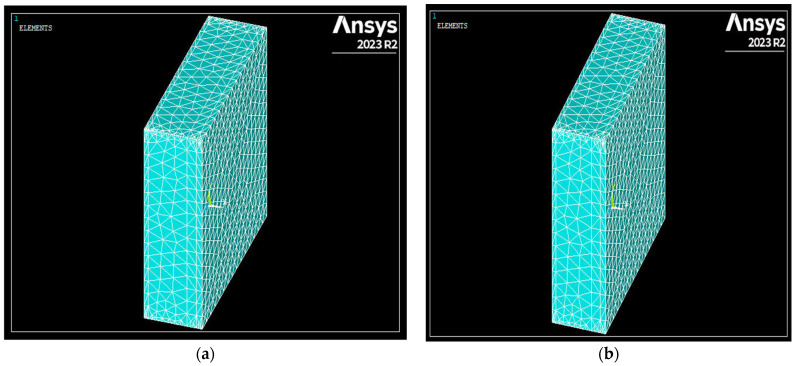
(**a**) Network view of the worst-performing building envelope among the alternative scenarios designed for Ankara province (Type 27). (**b**) Network view of the worst-performing building envelope obtained by ANSYS analysis using specimen SE45 (Type 31).

The alternative with the worst performance among the designed building envelopes is Type 27. This alternative was designed using brick as the construction material and wool as the insulation material in an internally insulated wall structure. When SE45-coded specimens were used as building materials in the wall components, the alternative scenario with the best performance was designated as Type 31. Figure 8 illustrates the network views of the wall temperature distributions obtained through ANSYS analysis for Type 27 and Type 31.

**Figure 9:**
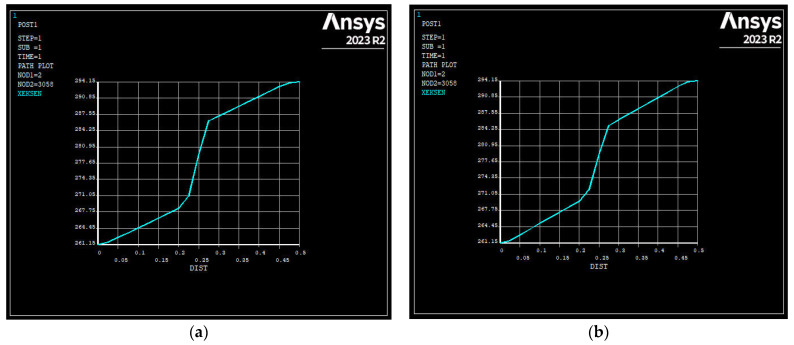
In case SE45-coded specimen is used as building material for sandwich wall type among alternative scenarios designed in Ankara province (**a**) Graphical representation of the temperature distribution of the best-performing building envelope (Type 16). (**b**) Graphical representation of the temperature distribution of the worst-performing building envelope (Type 15).

Then, the best and worst alternative scenarios were investigated when the SE45 specimen was used as the building material, and the building envelope was designed as a sandwich wall structure. The best alternative scenario was achieved with Type 16, where polyurethane was used as the insulation material, similar to Type 36, which previously showed the best performance among all alternative types. Similarly, the worst alternative scenario was determined with Type 15, where rock wool was used as the insulation material, similar to Type 27, which previously performed the worst among all alternatives. A graphical representation of the wall temperature distributions obtained through ANSYS analysis for Type 16 and Type 15 is shown in Figure 9.

## 4. Conclusions

Nowadays, due to ecological concerns and the non-renewability of oil resources, there is increasing attention toward the application and preparation of bio-based materials [73]. Polymer-based materials derived from living biological resources, such as trees and plants, are referred to as bio-based polymeric materials [74]. In efforts to protect ecosystems, environmental regulations are becoming more stringent, which is driving the use of renewable resources such as plant-based oils. Plant oils, which are lipids and fats containing triglyceride molecules, serve as a potential source of raw materials for the synthesis of high-functionality polyols. These polyols are widely used in the production of premium products, such as poly(ether urethanes) and polyurethanes. The highly effective polymers, monomers, and composite materials based on epoxidized plant oils can effectively replace analogous products made from epoxy resin. Vegetable oils, including sesame, corn, linseed, sunflower, castor, and soybean oils, have emerged as an attractive platform for chemical industries and academic research for producing various plastic-based materials due to their low cost, inherent biodegradability, and global availability [3].

In this study, only three different materials (epoxidized sesame oil, clay, and Seyitömer fly ash) were used to produce building-insulation samples. Fifteen samples were prepared with different proportions of the materials used [epoxidized sesame oil (30%, 35%, and 40%), clay (30%, 40%, 50%, 60%, and 70%) and Seyitömer fly ash (30%, 40%, 50%, 60%, and 70%)]. A total of 45 samples were obtained by producing three pieces each from all prepared samples. The produced samples were poured into molds. All samples were subjected to a pre-drying process at a temperature of 95 °C, which was obtained from preliminary trials. Finally, 15 samples were kept in the oven at 145 °C, and 15 samples at 165 °C and 15 samples at 185 °C at the same time intervals and made ready for the experiments. The heat conduction coefficient, mass, and compressive strength values of 45 materials produced were tested. Tensile strength values were calculated based on the compressive strength measurements. Among all the materials obtained experimentally, the material with the lowest thermal conductivity and mass (designated as SE45) was selected for technical evaluation as a building material with insulation properties. The theoretical modeling of this material was performed by considering the porosity and thermal conductivity characteristics of all materials. A deviation of approximately 5.88% was found between the theoretical modeling and experimental results. While the compressive strength does not meet the required standard, further research will be conducted to improve this value in future studies.

In the final part of the study, the energy performance of building exterior walls—namely, those with internally insulated, externally insulated, and sandwich structures—is investigated by analyzing the temperature distribution along the wall, taking into account the climatic conditions of Ankara (Turkey). To achieve this, 48 different alternative scenarios were prepared, combining traditionally applied building insulation materials with SE45-coded specimens as building materials. Using ANSYS software, the most efficient components in terms of temperature distribution along the wall thickness were identified.

## Figures and Tables

**Table 1:** The plant-sourced oils’ fatty acids with the highest number of esters.

Acid	Formula	Structure (C:DB)	Formula	Chemical Structure
Lycanic	C_18_H_28_O_3_	18:3	4-oxo-cis, trans, trans-11,13octadeca-trienoic	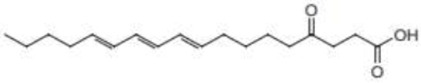
Vernolic	C_18_H_32_O_3_	18:1	12,13-epoxy-cis-9octadecenoic	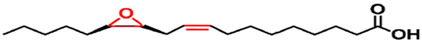
Ricinoleic	C_18_H_34_O_3_	18:1	9-12-hydroxy-cis-9octadece- noic	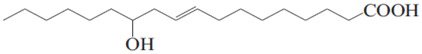
Erucic	C_22_H_42_O_2_	22:1	cis-13-dosocenoic	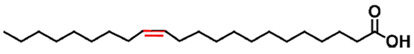
Alfa-eleosteafic	C_18_H_30_O_2_	18:3	cis, trans, trans-, 1113octadeca-trienoic	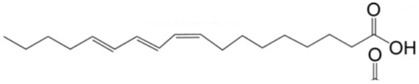
Linolenic	C_18_H_30_O_2_	18:3	cis-9,12,15-octadecatrienoic, cis	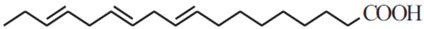
Linoleic	C_18_H_32_O_2_	18:2	Cis/9,12-15/octadecadienoic, cis	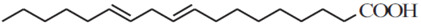
Oleic	C_18_H_34_O_2_	18:1	Cis/9/octadecenoic	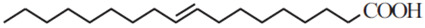
Palmitoleic	C_16_H_30_O_2_	16:1	Cis/9-hexadecenoic	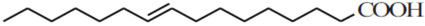
Lignoceric	C_24_H_48_O_2_	24:0	Tetracosanoic	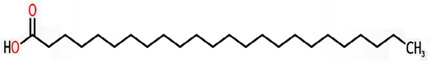
Behenic	C_22_H_44_O_2_	22:0	Docosanoic	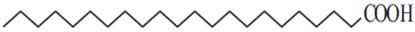
Arachic	C_20_H_40_O_2_	20:0	Eicosanoic	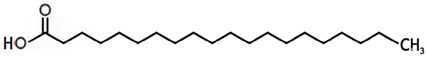
Stearic	C_18_H_36_O_2_	18:0	Octadecanoic	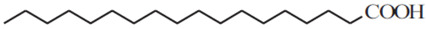
Palmitinic	C_16_H_32_O_2_	16:0	Hexadecanoic	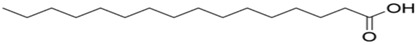
Myristitic	C_14_H_28_O_2_	14:0	Tetradecanoic	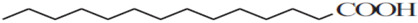
Lauric	C_12_H_24_O_2_	12:0	Dodecanoic	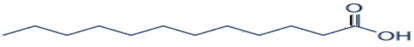

DB-shows the number of double bonds in the fatty acids’ chain and C-Carbon atoms’ a quantity.

**Table 2:** The research on composite materials sourced from plant oils.

Ref.	Results	Materials	Implementation
[36]	The hydrophilicity of the composite is improved due to the OH^−^ classes present in the grafted polymer.	Soybean oil (epoxidized) + cellulose powder (microcrystalline) + 1-methylimidazole + cis-1,2-cyclohexane-carboxylic anhydride	Cosmetics, food processing, pharmaceutical, and medical implementation
[37]	As a substitute crosslinker for epoxy, divinyl acrylic polymeric acid is synthesized from rosin acid. The addition of divinyl acrylic pimaric acid to epoxy resulted in reduced water absorption and volumetric swelling.	Soybean oil (methacrylated) + fibers of chicken feather + tert-butyl-peroxybenzoate + co-monomer	Household items (panels, frames, chairs, etc.)
[38]	The Young’s modulus increased by 400%, and the maximum tensile strength improved by 260%. The composite exhibited thermal stability up to 300 °C.	Linseed oil (epoxidized) + synthesized fillers + pripol1009 + DMAP	Bio-sourced thermoset materials, high-temperature implementations, membrane separation.
[39]	A nanocomposite with excellent thermal stability and strong antibacterial properties was developed.	Soybean oil (dried epoxidized) + DMAP + Cu2O NPs/Cu	Biomedical applications, anti-bacterial material
[40]	The addition of 10 weight percent starch results in a slight improvement in mechanical features. Increasing the starch content to 20 weight percent leads to approximately a 150% increase in Young’s modulus and tensile strength, while elongation at break decreases by 12%.	Linseed oil (epoxidized) + pripol-1009 + DMAP + starch (expanded)	Bio-sourced option, biocomposite, vinyl films
[41]	There is an increase in impact strength (37%), flexural modulus (2%), and tensile strain (4%)	Plant oils (epoxidized) + lignin powder (alkaline) + PLA	3 dimensional printing implementation
[42]	For dynamic loading, a silica-to-silanol ratio of 0.6 is found to be ideal, whereas a ratio of 2.0 is helpful for static applications.	Plant oils (epoxidized) + stearic acid + zinc oxide + silica + butadiene rubber + styrene butadiene rubber	Dynamic and static implementation
[43]	Strength, flexural modulus, Young’s modulus, and tensile strength all improved. The reduced amplitude of tan(δ) in the produced composite indicates enhanced interfacial adhesion between the fibers and the matrix.	Soybean oil (epoxidized and acrylated) + sisal preforms (short) + luperox P	Bio-composite materials
[44]	Higher non-homogeneity was observed in sheets made from distorted pellicles through β-radiography. Pristine pellicle sheets exhibited high tensile strength and fracture toughness.	Soybean oil (epoxidized and acrylated) + pristine sheet and cellulose pellicle (bacterial)	Composite materials for medical implementation
[45]	An improvement in fracture toughness, impact strength, and tensile strength was observed. When flax fiber was added, impact strength and cross-linking were enhanced, while epoxy methyl ricin oleate exhibited a higher storage modulus compared to castor oil (epoxidized).	Castor oil (epoxidized) + flax woven + sodium methoxide + methanol	Automotive and structural implementation
[46]	The reaction enthalpy was higher with 1%/3% chitosan loading compared to 5% loading. Chitosan’s low crosslinking density was indicated by its swelling ratio.	Soybean oil (epoxidized) + salicylic acid + chitosan	Composites, curing agents
[47]	Trimer formation occurs due to the combination of anhydride and epoxy groups. The increase in tensile strength is attributed to H_2_ bonding.	Soybean oil (epoxidized) + maleic anhydride tung oil and Tung oil and soybean oil (epoxidized) + maleic anhydride	Self-healing implementation

**Table 3:** The fatty acid composition of selected plant oils, including sesame oil [%].

Acid Name	Sesame	Com	Canola	Olive	Rapeseed	Sunflower	Grape	Flaxseed	Rice Oil	Peanut	C:DC
Newomc	0	0	0.12	0	0.12	0	0	0	0	0	24:1
Lignoceric	0.08	0.17	0.13	0.05	0.16	0.24	0.16	0.12	0.42	1.39	24:0
Elucic	0	0	0	0	0.07	0.04	0.05	0	0.06	0.21	22:1
Behenic	0.13	0.12	0.25	0.11	0	0.78	0.41	0.16	0.26	2.62	22:0
-	0.19	028	0.98	029	126	0.16	0.19	0.10	0.53	1.15	20:1
Arachidic	0.62	0.41	0.53	0.41	0.58	0.24	0.27	0.16	0.87	1.47	20:0
Linolenic	0	0.8	6.01	0.63	8.08	0.05	0.85	52.65	0.93	0.5	18:3
Linoleic	43.19	56.31	18.66	6.17	18.47	5825	60.45	15.85	30.26	19.96	18:2
Oleic	40.05	28.38	51.35	77.86	64.47	30.4	25.39	20.38	44.93	59.26	18:1
Stearic	5.77	1.78	2.37	3.15	193	3.27	3.8	4.35	2.06	3.53	18:0
Margarooleic	0	0.03	0.06	0.13	0.09	0.03	0.04	0	0.02	0.05	17:1
Margaric	0.04	0.06	0.06	0.07	0.05	0.04	0.05	0.06	0.04	0.07	17:0
Palmitooleic	0.15	0.13	0.25	0.81	024	0.13	0.15	0.07	4.06	0.13	16:1
Palmitic	9.32	11.06	12.53	1027	4.05	6.62	8.06	5.87	15.23	9.47	16:0
Myristic	0.02	0.03	0.29	0	0.05	0.07	0.07	0	0.26	0.03	14:0

**Table 4:** Technical properties of sesame oil.

Features	Unit	Value
Viscosity	[cSt (mm^2^/s) at 40 °C]	36
Acidity	[mg·KOH/g.oil]	4.6
Pour point	[°C]	−9
Loss tangent at 50 Hz	[%]	18
Conductivity at 1 mHz	[pS/m]	1041
Permittivity at 1 kHz	[cSt (mm^2^/s)]	3.04
Average Breakdown Voltage	[kV]	14.3
Total saturation	[%]	13
Total unsaturation	[%]	87

**Table 5:** The disadvantages and advantages of various epoxidation strategies.

Strategies	Catalysts	Reactants	Disadvantages	Advantages
Polyoxometalate catalysts methodology	Polyoxometalates	H_2_O_2_	Difficult separation	Restricted side reactions
			No reusability	Mild circumstances quickly
			Catalysts’ deactivation	Full conversion
			Instability	High selectivity
Chemoenzymatic catalysis	Biocatalyst	H_2_O_2_, Fatty acid	No reusability	Restricted side reactions
			High cost	Mild circumstances
			Less stability	Enhanced chemoselectivity
				High stereoselectivity and regioselectivity
				High Yield
Metal mediated Differential catalysis	Metal sourced catalysts	H_2_O_2_, Carboxylic acid	Less regioselectivity	Less costs
			Toxicity	Restricted oxirane ring opening
			Less stereoselectivity	Limit side reactions
				Maximum selectivity
				High conversion and yield
Heterogenous Catalysis	Acidic ion exchange resins	H_2_O_2_, Carboxylic acid	High costs	Easy produce separation
			Less stereoselectivity	Restricted side reactions
			Less regioselectivity	More selectivity
				Great yield
				Green and clean
Homogenous Catalysis	Mineral acid	H_2_O_2_, Carboxylic acid	Thermal runway	Less cost
			Side reactions	Great yield,
			Corrosion	
			Decreased Selectivity	

**Table 6:** The characteristics of the fly ash and clay used in this study.

	SiO_2_	Fe_2_O_3_	Al_2_O_3_	CaO	Na_2_O	K_2_O	SO_3_	MgO	Ignition Loss	(Unknown)
	wt%	wt%	wt%	wt%	wt%	wt%	wt%	wt%	wt%	wt%
Fly ash (Seyithan)	33.54	4.43	15.27	34.62	0.18	1.42	2.56	2.65	5.18	0.19
Clay	48	16.80	16.24	0.42	1.10	0.70	0.12	0.56	16.00	0.06

**Table 7:** The properties of epoxidized sesame oil [51,56].

Features	Unit	Value
Epoxidation extent	[%]	96.2
Moisture content	[%]	7.57
Density	[g·mL^−1^]	1.04
Iodine index	[gI2/100 g]	7.67
Acid index	[mg·(NaOH)·g^−1^]	18.7
Acid number	[mg·(KOH)/g^−1^]	8.11
Oxirane oxygen	[%(p/p)]	1.775
Thermal conductivity coefficient	[W/mK]	0.82
Process parameters with Performic acid		
Reaction yield	[%]	28.53
Carboxlic acid/C=C molar ratio	[mol/mol]	0.8:1
Glycols concentration	[mol/IOO g oil]	0.03
Conversion	[%]	90.7
Relative conversion to oxirane	[%]	84.6
Oxirane O_2_ content	[%]	5.5
Selectivity	[%]	93.2
Epoxy number	[mol/100 g oil]	0.34
Stirring speed	[wt%-rpm]	700
Temperature	[°C]	80

**Table 8:** The sample codes for the experiments.

Sample Codes Range from ‘SE1’ for ‘1’ to ‘SE45’ for ‘45’.									
Exp. Clay -Fly Ash	145 °C			165 °C			185 °C		
	30%	35%	40%	30%	35%	40%	30%	35%	40%
70–30%	1	2	3	4	5	6	7	8	9
60–40%	10	11	12	13	14	15	16	17	18
50–50%	19	20	21	22	23	24	25	26	27
40–60%	28	29	30	31	32	33	34	35	36
30–70%	37	38	39	40	41	42	43	44	45

**Table 9:** Mathematical expressions of elementary cell components’ thermal resistances.

$R_{1K}=\frac{D}{k_{K}\left(D^{2}-L^{2}\right)}$	$2R_{2K}=\frac{\left(D-L\right)}{k_{K}\left(L^{2}-I^{2}\right)}$	$2R_{3K}=\frac{\left(D-L\right)}{k_{K}I^{2}}$
	$R_{2U}=\frac{L}{k_{U}\left(L^{2}-I^{2}\right)}$	$2R_{3U}=\frac{\left(L-I\right)}{k_{U}I^{2}}$
		$R_{3G}=\frac{I}{k_{G}I^{2}}$

**Table 10:** Technical values of the fly ash and clay materials used in the experiments.

Material	Density (g/cm^3^)	Thermal Conductivity Coefficient (W/mK)
Seyitömer chimney bottom fly ash	2.100	0.097
Clay	1.527	1.002

**Table 11:** The design of alternative scenarios with SE45 code sample and traditional building insulation materials for three different wall structures.

	Type	Insulated Wall Structure	External Plaster	Building Material	Insulation Material	Building Material	Internal Plaster
			(From Outdoor to Indoor)				
GROUP A	1	Sandwich	+	gas concrete	EPS	gas concrete	+
	2	Sandwich	+	gas concrete	XPS	gas concrete	+
	3	Sandwich	+	gas concrete	rock wool	gas concrete	+
	4	Sandwich	+	gas concrete	polyurethane	gas concrete	+
	5	Sandwich	+	bims	EPS	bims	+
	6	Sandwich	+	bims	XPS	bims	+
	7	Sandwich	+	bims	rock wool	bims	+
	8	Sandwich	+	bims	polyurethane	bims	+
	9	Sandwich	+	brick	EPS	brick	+
	10	Sandwich	+	brick	XPS	brick	+
	11	Sandwich	+	brick	rock wool	brick	+
	12	Sandwich	+	brick	polyurethane	brick	+
	13	Sandwich	+	SE45	EPS	SE45	+
	14	Sandwich	+	SE45	XPS	SE45	+
	15	Sandwich	+	SE45	rock wool	SE45	+
	16	Sandwich	+	SE45	polyurethane	SE45	+
GROUP B	17	Internal	+	gas concrete	EPS	-	+
	18	Internal	+	gas concrete	XPS	-	+
	19	Internal	+	gas concrete	rock wool	-	+
	20	Internal	+	gas concrete	polyurethane	-	+
	21	Internal	+	bims	EPS	-	+
	22	Internal	+	bims	XPS	-	+
	23	Internal	+	bims	rock wool	-	+
	24	Internal	+	bims	polyurethane	-	+
	25	Internal	+	brick	EPS	-	+
	26	Internal	+	brick	XPS	-	+
	27	Internal	+	brick	rock wool	-	+
	28	Internal	+	brick	polyurethane	-	+
	29	Internal	+	SE45	EPS	-	+
	30	Internal	+	SE45	XPS	-	+
	31	Internal	+	SE45	rock wool		+
	32	Internal	+	SE45	polyurethane		+
GROUP C	33	External	+	-	EPS	gas concrete	+
	34	External	+	-	XPS	gas concrete	+
	35	External	+	-	rock wool	gas concrete	+
	36	External	+	-	polyurethane	gas concrete	+
	37	External	+	-	EPS	bims	+
	38	External	+	-	XPS	bims	+
	39	External	+	-	rock wool	bims	+
	40	External	+	-	polyurethane	bims	+
	41	External	+	-	EPS	brick	+
	42	External	+	-	XPS	brick	+
	43	External	+	-	rock wool	brick	+
	44	External	+	-	polyurethane	brick	+
	45	External	+	-	EPS	SE45	+
	46	External	+	-	XPS	SE45	+
	47	External	+	-	rock wool	SE45	+
	48	External	+	-	polyurethane	SE45	+

**Table 12:** Technical properties of materials used in ANSYS analysis [7,8,9,10,11].

Material	Exter. Plast.	Building Materials				Insulation Materials				Inter. Plast.
		Gas Concrete	Bims	Brick	SE45	EPS	XPS	Stone Wool	Polyurethane	
Thickness (m)	0.02	0.20	0.20	0.20	0.20	0.05	0.05	0.05	0.05	0.03
Thermal conductivity (W/mK)	0.85	0.18	0.23	0.62	0.256	0.035	0.034	0.041	0.030	1.40
Density (g/cm^3^)	1.400	0.82	0.77	1.80	1.30	0.024	0.022	0.215	0.033	2.000

## Data Availability

The data presented in this study are available upon request from the corresponding author. The data are not publicly available due to the complexity of the calculations.
